# Assessing and Forecasting Fatigue Strength of Metals and Alloys under Cyclic Loads

**DOI:** 10.3390/ma17071489

**Published:** 2024-03-25

**Authors:** Andrey Kurkin, Alexander Khrobostov, Vyacheslav Andreev, Olga Andreeva

**Affiliations:** 1 Department of Applied Mathematics, Nizhny Novgorod State Technical University, n.a. R.E. Alekseev, 603155 Nizhny Novgorod, Russia; 2 Department of Nuclear Energy and Technical Physics, Nizhny Novgorod State Technical University, n.a. R.E. Alekseev, 603155 Nizhny Novgorod, Russia; khrobostov@nntu.ru (A.K.); vyach.andreev@mail.ru (V.A.); andreevaov@gmail.com (O.A.)

**Keywords:** fatigue limit, fatigue life, cyclic loads, S-N curve, fatigue analysis, data analysis

## Abstract

Within the scope of this research, patterns of changes in the fatigue life and limit of metals under cyclic stress were identified and the most informative parameters were determined as the basis for developing a method for the universal transformation of experimental data on the fatigue of metals and alloys for their subsequent generalization. Experimental data on metal fatigue, obtained by a large number of authors for a wide range of grades of steels and alloys, under the influence of various combinations of factors, were systematized. A generalized dependence of the recalculated parameters of fatigue life and limit was obtained, its characteristics were assessed, and a sensitivity analysis was performed, confirming the universal nature of the obtained dependence. A system of parameters has been proposed making it possible to consider and forecast high-cycle fatigue processes for a wide range of metals and alloys, under the conditions of various combinations of operating factors, from unified positions and a more general point of view.

## 1. Introduction

Over a fairly long period of studying the fatigue phenomenon [1,2,3,4], extensive data have been accumulated on test metals and other structural materials under cyclic loading. The destruction of metals under cyclic loading is a statistical process. At the same level of applied stresses, the destruction of the material or some recorded parameter reaching a critical value, for example, the degree of damage to the surface microstructure, can occur over a different number of cyclic loadings [5,6].

In the works of various materials scientists, attempts have been made to introduce a fatigue resistance integral indicator—fatigue curve angle of inclination—to the abscissa axis (number of stress cycles) in the high-cycle fatigue region of the fatigue curve in the logarithmic scale. This indicator is called a structurally sensitive indicator of fatigue resistance, taking into account its relationship with the damage to the surface microstructure of a cyclically loaded metal or alloy [7].

However, a single fatigue indicator does not provide a complete picture of the material’s ability to resist fatigue and the position of the fatigue curve on the logarithmic scale. Taking into account the fact that a single fatigue indicator is not enough. It is also required the coordinates (value of stress and number of stress cycles) of any point belonging to the high-cycle region of the fatigue curve. Only consideration of these entire characteristics makes it possible to unambiguously predict the fatigue curve position and to correctly select metallurgical, structural, technological, and operational factors, that provide the greatest fatigue strength. The fatigue curve angle of inclination to the number of stress cycles’ axis, justifying its name—the integral indicator—is a characteristic for the entire section of the high-cycle region of the fatigue curve. Any arbitrary point on a fatigue curve is a complicated object if it is necessary to compare several fatigue curves with each other. However, let us assume that in all fatigue curves there is a special point, which, despite a large difference in the absolute values of its coordinates, in its physical meaning corresponds to the same “special” state of metals under cyclic loading. At this “knee-point” [8] or the breaking point of the fatigue curve in the high-cycle region, a transition occurs from one controlling mechanism of damage accumulation to another. Above this point (if the fatigue curve will be described along the *y*-axis—according to the magnitude of the load that cyclically acts on the material) there is an area in which damage to the material occurs mainly due to the growth and propagation of micro cracks, which ultimately form a crack of a critical size. Below this point, the controlling mechanism of the process of metal destruction under cyclic loading will be the fusion of micropores. Processing the test results and presenting them in a logarithmic coordinate system makes it possible to observe, in the vicinity of this point, two characteristic sections of the fatigue curve in the high-cycle region—the left (steeper) and right (flatter, almost horizontal) branches of the fatigue curve. The fatigue limit coordinates are considered to be fatigue resistance parameters. Since these indicators correspond to similar states of the material under cyclic loading, their comparison with each other for different fatigue curves is justified.

**Figure 1:**
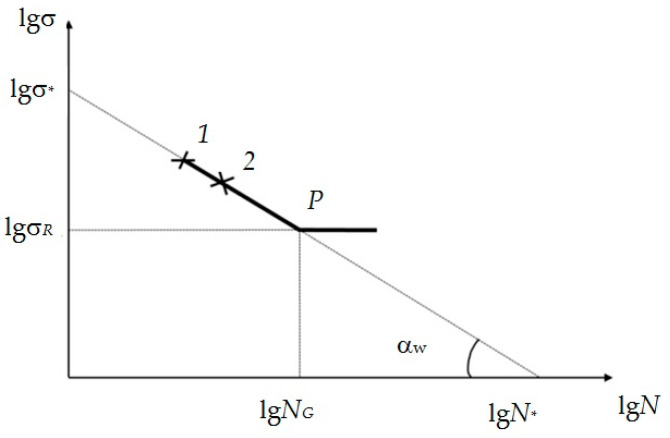
Schematic representation of fatigue curve high-cycle region in a logarithmic scale: *P*—fatigue limit (breaking point (“knee point” [8]) of the fatigue curve); lg*N*—logarithm of stress cycle number; lgσ*_R_*—logarithm of stress corresponding to the fatigue limit; lg*N_G_* is the abscissa of the fatigue curve breaking point (the logarithm of the number of stress cycles corresponding to the fatigue limit and the fatigue curve transition to an almost horizontal section); α*_w_* is a structure-sensitive parameter of metal fatigue in a logarithmic coordinate system; lgσ_*_, lgN_*_—conditional, physically unrealizable values of stress and number of cycles logarithms at which the straightened fatigue curve intersects the coordinate axes 1 and 2—experimental points corresponding to the destruction of the objects under study after a certain number of stress cycles *N*1 and *N*2 at stress levels σ_1_и σ_2_; lgσ = lgσ_*_ − tg(α*_w_*)lg*N*—equation of the left branch of the fatigue curve.

The high-cycle region of the fatigue curve in logarithmic scale (shown in Figure 1 [9]) represents two straight-line segments intersecting at the breaking point which corresponds to the fatigue limit. The left, steeper section is characterized by the angle of inclination to the number of stress cycles’ axis. The right one, in most cases, is considered horizontal.

Fatigue data systematization to ensure the possibility of joint processing and obtaining some generalized characteristics of cyclic load impact on a structural material is of great interest. This operation, if successfully implemented, would reduce the volume of upcoming tests due to their better planning and provide the possibility of a joint analysis of all previously obtained experimental data.

The main goal of the current study is to summarize the experimental information on the fatigue of metals and alloys accumulated to date. To do this, it is required (1) to develop a procedure for converting experimental information, obtained from different data sources, on metal fatigue, which allows us to generalize experimental data on the high-cycle fatigue of metals, and (2) to obtain a generalized dependence of fatigue indicators. Following this, it becomes possible to develop a method for predicting fatigue indicators based on this generalized relationship and evaluate its accuracy by comparing it to experimental results of fatigue tests and other methods of predicting high-cycle fatigue indicators.

## 2. Materials and Methods

### 2.1. Data Sources and Systematization of Fatigue Information

Traditionally, for the graphical presentation of fatigue tests results, it is customary to use the relationship between the applied stress (usually a specified value) and the experimentally obtained number of stress cycles, corresponding to either the destruction of the sample or achieving a main crack of a certain length. If the sequence of the experiment is to be literally interpreted, load is an argument, and the number of cycles is a function.

Fatigue life and limit values vary widely depending on the combination of metallurgical, structural, technological, and operational factors under which cyclic loading occurs. The following pattern can be traced in various studies: the steeper the slope of the fatigue curve in the high-cycle region, the lower the fatigue life of parts at stresses exceeding the fatigue limit and, as a rule, the lower the fatigue limit [10,11,12,13].

The main sources of experimental data for this research are experimental results obtained in [13,14], objectively limited in volume and range of variation of operating factors, as well as the results of experimental determination of fatigue life and limit values [15]. 

The purpose of the experimental data systematization procedure was to determine what possible “states” the factors influencing the fatigue curve position were located in for each experimental result, which was used to construct a model of metal and alloy behavior under cyclic loading. To transform the empirical data into an information model, it was also necessary to pre-assign a certain numerical value (normalization factor) to each possible “state” of the factor under consideration. In addition, when performing such a transformation of qualitative data into quantitative data, it is much easier to carry out systematization in order to determine the frequency of the presentation of a particular “state” in the data under consideration. As a result of the analysis of all of the selected initial data, the following were identified:

- 204 different grades of steels, alloys, and pure metals;
- 17 different loading schemes, in which the cyclic loading of samples and full-scale part loading occurred;
- 54 different test environments;
- 46 temperature modes;
- 4 characteristic cross-sectional shapes of cyclically loaded samples or parts;
- 81 different heat treatment modes of the materials of laboratory samples and parts.

In order to ensure the possibility of using the collected data in a single system, a normalization factor was applied to each value [15]. For example, the influence of the stress cycle frequency was taken into account using the CF coefficient. The frequency of the stress cycle in the data used [14] ranges from 2.5 Hz to 20,000 Hz. The normalization factors for numerical values were calculated by Equation (1):

CF = (w − 10,001.25)/9998.75,(1)

where w is the frequency of the applied stress cycles when testing laboratory samples or parts for fatigue. The use of a neural network to predict fatigue parameters involves a stage for encoding the information about operating factors, the combination of which causes cyclic loading of a sample or a part. To perform the coding, a procedure similar to the coding procedure when constructing an interpolation model based on a fractional factorial experiment, was used. Here, discrete and continuous factors were distinguished. The following factors were considered as discrete ones: the grade of steel or alloy, the sample shape, the test environment, the surface treatment method, the loading pattern, and the heat treatment mode. The test temperature, the frequency of the acting load, the characteristic size, and the surface roughness were considered to be continuous factors. The main level of the factor and its range of variation were determined, which was a symmetrical interval around zero. Thus, the coding procedure made it possible to replace the natural factor values with coded values within a −1 to +1 range; the main level corresponding to 0. As an example of such formulas, the formula to encode information about the frequency of the applied stress cycles is listed in the article. In this case, the CF coefficient zero value corresponds to a 10,001.25 Hz frequency value; there were no such experimental data in the database. The nearest lower value of the operating load frequency at which fatigue tests were carried out is 10,000 Hz (12 such fatigue curves were considered as part of the generalized dependence) and the nearest higher value is 10,600 Hz (1 such curve was considered as part of the generalized dependence) [14].

**Figure 2:**
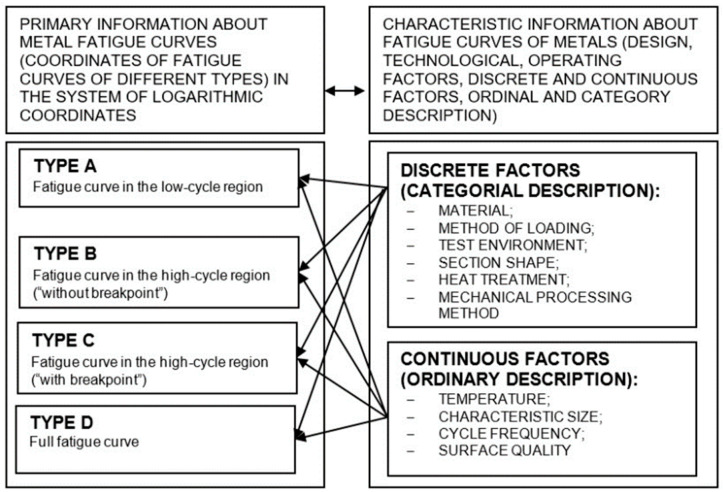
Generalized structure of data on fatigue resistance indicators of metals and alloys.

**Figure 3:**
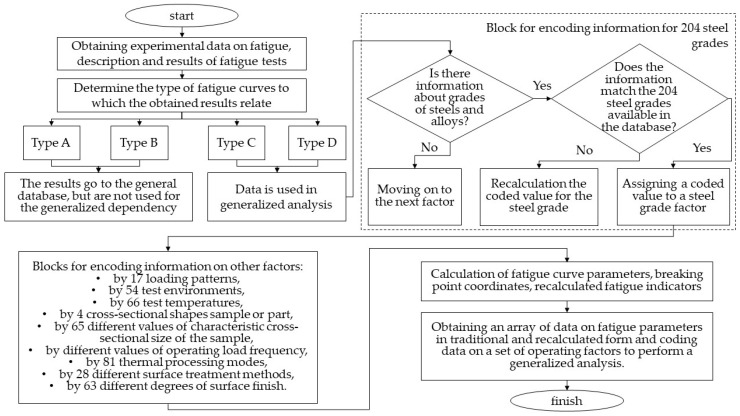
Diagram of the procedure for systematizing experimental data.

The variety of combinations of operating factors did not allow the use of accumulated data without preliminary structuring, and subsequent transformation of the obtained experimental data for the purpose of their subsequent effective use. To perform qualitative factor analysis, it was necessary to develop a system for storing and studying the information on test conditions and quantitative characteristics of fatigue curves. Figure 2 shows the generalized structure of the data obtained and Figure 3 shows a diagram of the procedure for systematizing experimental data. 

Figure 3 shows, in detail, the block of information systematization by steel grades. A similar procedure was followed for the remaining nine factors listed in “Blocks for encoding information for other factors”.

The cost of these experimental data is enormous, since determining the position of the fatigue limit requires experimentally determining the position of the fatigue curve’s high-cycle region [16,17,18]. Carrying out such experimental work requires high time and material costs, both for producing laboratory samples, with high demands placed on the accuracy of their manufacture and processing, and for conducting the experiments themselves, with their high implementation costs. On the other hand, it is practically impossible to repeat published experimental works in the same volume and under the same conditions. However, despite these high costs, already published experimental data are of little practical use, at least in the sense that the data were obtained with random combinations of operating factors that were determined by a specific problem solved by a particular researcher [19]. Despite being unique (for example, the cyclic stress of metals in mercury), the data do not often make it possible to conduct joint consideration and analysis due to incomplete descriptions of operating factor sets.

**Figure 4:**
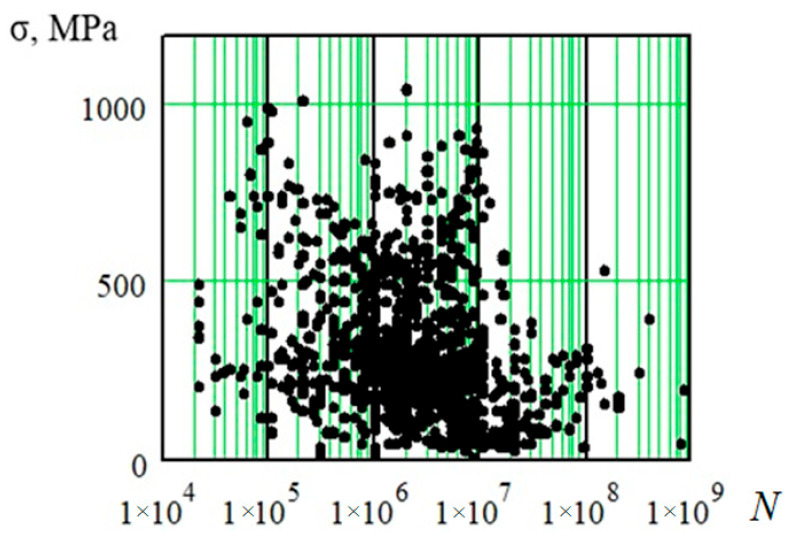
Breaking point position of metal fatigue curves in high-cycle region (σ—stress, MPa; lg*N*—logarithm of stress cycles number (number of cycles to failure)).

These kinds of experimental results are commonly used to find the data closest to the test conditions to experimentally determine fatigue resistance indicators and their subsequent “refinement”, based on expert assessments, for extension to areas determined by the conditions of the forecasting problem being solved [20,21,22]. A joint graphical representation of two high-cycle fatigue parameters for all studied curves (1179 fatigue curves with fatigue limits in the high-cycle region) is shown in Figure 4 [9]. This figure shows the breaking point (physical fatigue limits) positions of metals and alloys on fatigue curves and their experimental information is analyzed in the article.

All considered points (Figure 4 contains 1179 fatigue limits of fatigue curves) lie chaotically in a certain area, which can only be characterized by an approximate description of its boundaries. This “cloud” contains fatigue curve breaking points obtained for various grades of steel under cyclic loads of various natures, which are influenced by a wide range of factors. The location of the breaking points of a particular fatigue curve depends on the set of acting factors, but with such a graphical representation of the experimental results, it is difficult to obtain quantitative relationships between the position of the breaking point and the set of acting factors.

### 2.2. Data Exploration Based on Data Normalization

In the current study, the main “tool” and condition for obtaining an explicit generalized dependence of normalized fatigue parameters was the normalization procedure; that is, the procedure for obtaining relative (recalculated) indicators of fatigue resistance characterizing the high-cycle region of the fatigue curve. Three indicators, which were obtained for each fatigue curve based on the experiments, had to be compared with each other—the fatigue limit, the abscissa of the fatigue curve breaking point in the high-cycle region or, in other words, the number of cycles at the fatigue limit and the slope of the fatigue curve’s left branch for presenting it in a logarithmic scale. Two of the three indicated values are dimensional ones that vary over a wide range. For example, the range of changes in fatigue limits for the fatigue curves considered as part of the generalized dependence is from 10 MPa to 1050 MPa. And the number of cycles at the fatigue limit ranges from 2 × 10^4^ to 8 × 10^8^ stress cycles. The angle of inclination of the tangent of the fatigue curve to the number of stress cycles axis also varies in a wide range from 0.01 to 7.692. In such cases, in order to compare these quantities, a normalization procedure is advised which simultaneously considers relative and dimensionless quantities. Experimental data generalization is widely used in engineering practice in order to obtain dependencies characterizing the occurrence of certain physical processes in such systems. For example, criterion empirical relationships between dimensionless parameters are widely used to assess the efficiency of heat removal (heat transfer) within a wide range of boundary conditions and geometric formulations [23]. Such equations represent approximation by power function the dependence of one dimensionless number, for example, the Nusselt similarity criterion, on other dimensionless numbers (for example, on the Grashof number for free convection and on the Reynolds number for forced flow, etc.). A similar approach is used in the field of materials science (in particular, to rationalize the graphical presentation of fatigue test results [1,8] in hydraulics and in heating engineering [24]). Provisions of similarity theory, dimensional analysis, and the method of characteristic scales, which represent different areas of generalized analysis, are used in most methods [24]. According to [24], such a transformation of information regarding test results is a variant of a special method to reduce the difficulties associated with the multiplicity of the internal and external parameters of the task. Transforming the problem parameters with further transition to their dimensionless analogues makes it possible to obtain forms of dependencies between the quantities under study that are more convenient for analytical description, to predict the behavior of the system under study, and, after restoring the traditional form of the parameters from their transformed analogues, to formulate the obtained forecast guidelines for a specific engineering system. In the current work, the recalculated fatigue parameters were analyzed during the procedure of selecting and evaluating the best form according to the fatigue parameters of the transformed indicators and various forms of normalized coefficients.

### 2.3. Generalization of Heterogeneous Information on Metal Fatigue

Even if the angle of inclination of the right branch is different to zero, the angle of inclination of the tangent for this section differs to a lesser extent, as a rule, by an order of magnitude compared to the angle of inclination of the tangent of the left branch. Obviously, to predict the fatigue curve position in the high-cycle region, when representing it in logarithmic coordinates, it is necessary to know three parameters: the fatigue limit σ_R_, the number of stress cycles corresponding to the breaking point on the fatigue curve N_G,_ and the slope of the left branch of the fatigue curve to the axis of the number of stress cycles tgα*_W_*.

Initially, it is necessary to identify certain variables, their relationship simplifying the task of predicting the fatigue curve position. Despite the absence of explicit functional connections between relevant fatigue curve parameters in the high-cycle region (σ_R_, N_G_, tgα*_W_*), they should nevertheless exist [2,25]. This is, in particular, suggested by the fact that, under certain conditions, a larger slope of the fatigue curve corresponds to a smaller fatigue limit and vice versa. These dependencies were initially identified by limited experimental data.

When performing the comparison, due to the objective limitation of data on breaking point coordinates on the fatigue curves and small areas of change acting under cyclic loading factors, the relationship between relevant recalculated parameters was obtained in a first approximation, namely in the form of a rectilinear approximation of the points obtained by transforming the experimental data. In analytical form, the relationship between the recalculated parameters (in [26] a relationship was identified only between the recalculated strength and the fatigue curve slope tangent in the high-cycle region) is represented by Equation (2).

lg((σ_R_/σ_*_) × 100) = 3.4590 − 1.9432 lg(tgα*_W_* × 100). (2)

### 2.4. “Tools” to Select the Best Shape for Normalized Fatigue Parameters

Traditionally referred to as part of data preprocessing, normalization can be performed using various analytical expressions for the processed quantities [8,27]. This can be normalization with a focus on extreme values accepted by the quantities under study; for example, by normalizing each variable to the range of the dispersion of its values (Equation (3)):

(3) $${\tilde{x}}_{i}=\frac{x_{i}-x_{i\min}}{x_{i\max}-x_{i\min}},$$

where ${\tilde{x}}_{i}$ is the random element of normalized data, *x_i_* is the corresponding data element source; *x_i_*_max_ − *x_i_*_min_ is the range of scattering values of input data elements.

A more reliable procedure can be carried out with a focus on typical or statistical characteristics of the data, such as mean and variance (Equation (4)):

(4) $${\tilde{x}}_{i}=\frac{x_{i}-{\bar{x}}_{i}}{\sigma_{i}},$$

where ${\bar{x}}_{i}=\frac{1}{n}\sum_{i=1}^{n}x_{i}$ is the average of all data elements, $\sigma_{i}=\sqrt{\frac{1}{n-1}\sum_{i=1}^{n}{\left(x_{i}-{\bar{x}}_{i}\right)}^{2}}$ is the standard deviation of the value *x_i_*.

For the current study, data normalization for subsequent analyzes was performed separately for each fatigue curve. Among possible relative variables characterizing the fatigue curve breaking point position, the following variables were considered: σ_R_/σ_*_, (σ_*_ − σ_R_)/σ_*_ to characterize the breaking point along the stress axis and N_G_/N_*_, (N_*_ − N_G_)/N_*_ to characterize the breaking point along the axis of the number of cycles. Analysis of the spread of experimental data in a joint study of the indicated relative characteristics of the breaking points on the fatigue curve depending on tgα*_W_* made it possible to select for further research, based on the larger value of the correlation coefficient, the first of the above pairs of variables, namely σ_R_/σ_*_ and N_G_/N_*_. That is, options for presenting normalized data according to Formula (3), in which *x_i_*_min_ is assumed to be equal to zero [8,27].

Taking into account the obtained results, relative characteristics were introduced: a relative characteristic of the metal fatigue curve position in the high-cycle region along the ordinate axis σ_R_/σ_*_ (in this context it can also be called the recalculated strength) and the other relative characteristic, obtained similarly, being N_G_/N_*_ (along the abscissa axis). The latter can be called recalculated fatigue life, corresponding to the fatigue curve breaking point in the high-cycle region.

To quantify the efficiency of converting information on metal fatigue, correlation coefficient calculation results between data sets and calculation of the conditional entropy were used. In the latter case, only quantitative assessment of the connection between data sets is assumed, without assessing these connection parameters.

A correlation coefficient is used to determine whether there is a relationship, for example, between two properties represented by two data sets. To calculate the correlation coefficient, the following Equation (5) was used.

(5) $$k=\frac{Cov(X,Y)}{\sigma_{x}\sigma_{y}},$$

where σ*_x_*, σ*_y_* are the standard deviations corresponding to random variables *X* and *Y*, *Cov*(*X*,*Y*) is the covariance or the average of the products of deviations for each pair of data points (Equation (6)):

(6) $$\mathrm{Cov}(X,Y)=\frac{1}{n}\sum_{j=1}^{n}\left(x_{j}-\mu_{x})(y_{j}-\mu_{y}\right),$$

where µ*_x_* and µ*_y_* are the sample means of the compared data sets and n is the sample size [28,29].

The correlation coefficient (k, Formula (5)) was used to determine whether there is a relationship between the compared values, as well as whether this relationship is direct or inverse. It was important to evaluate if the recalculated fatigue indicators are related to each other in the same way as the fatigue indicators were related to each other before the normalization transformation was used. Experimental data show that such a relationship is best seen between the angle of inclination of the tangent of the fatigue curve to the number of stress cycles axis and the fatigue limit. The steeper the left branch of the fatigue curve which is located in the logarithmic coordinate system, the smaller the fatigue limit is. The relationship between these fatigue indices and the abscissa of the fatigue curve breaking point in the logarithmic coordinate system based on the selected fatigue curves is not so obvious. At the same time, correlation analysis made it possible to identify such a relationship between all three fatigue indicators when comparing them with each other after transformation in order to obtain their reduced analogues. 

Another “tool” for selecting a regression model was the coefficient of approximation reliability (coefficient of determination) (K), a measure of how well the selected model fits in terms of explaining the variation in the selected variable. Generally, the coefficient of determination is considered to be the main indicator reflecting a measurement of the regression model quality.

## 3. Results

**Figure 5:**
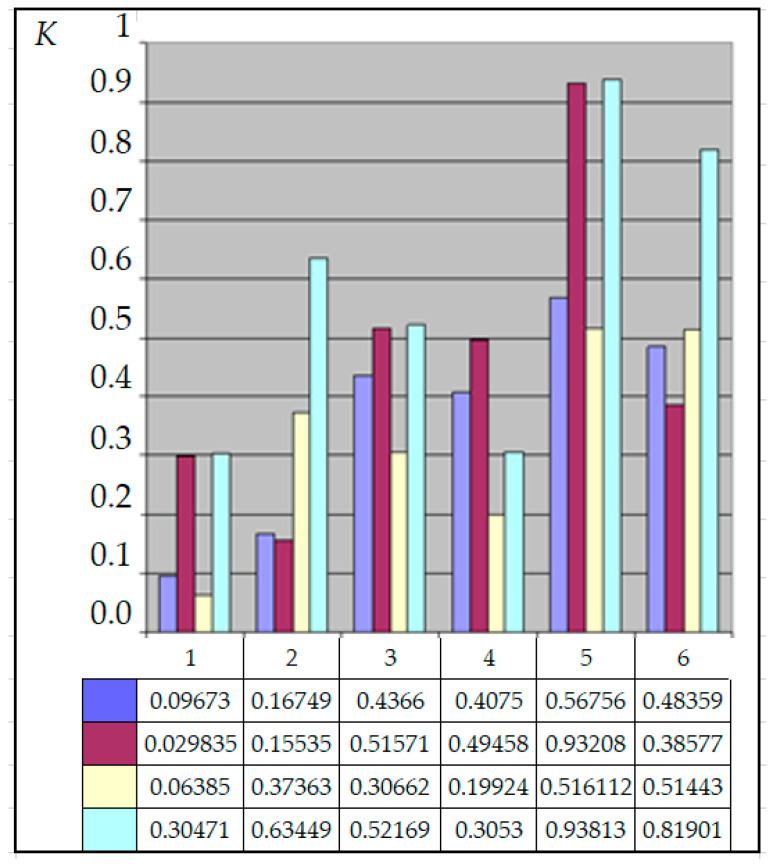
Evaluation of the relationship between the fatigue limit and the number of stress cycles corresponding to the fatigue curve breaking point.

**Figure 6:**
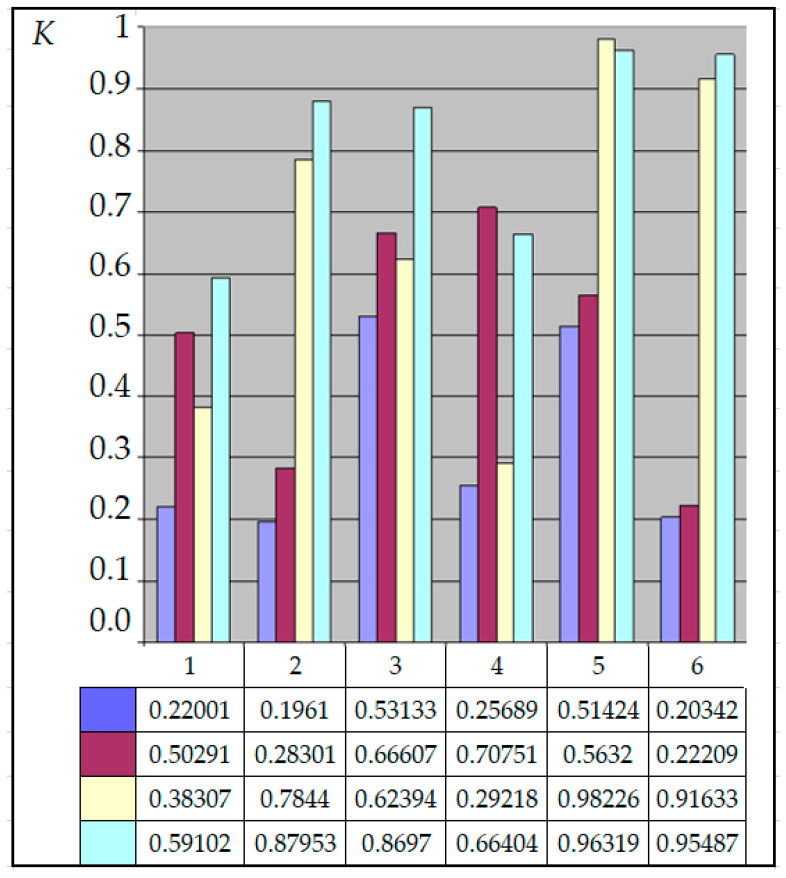
Estimation of the relationship between the fatigue limit and the angle of inclination of the tangent of the fatigue curve to the number of stress cycles axis.

**Figure 7:**
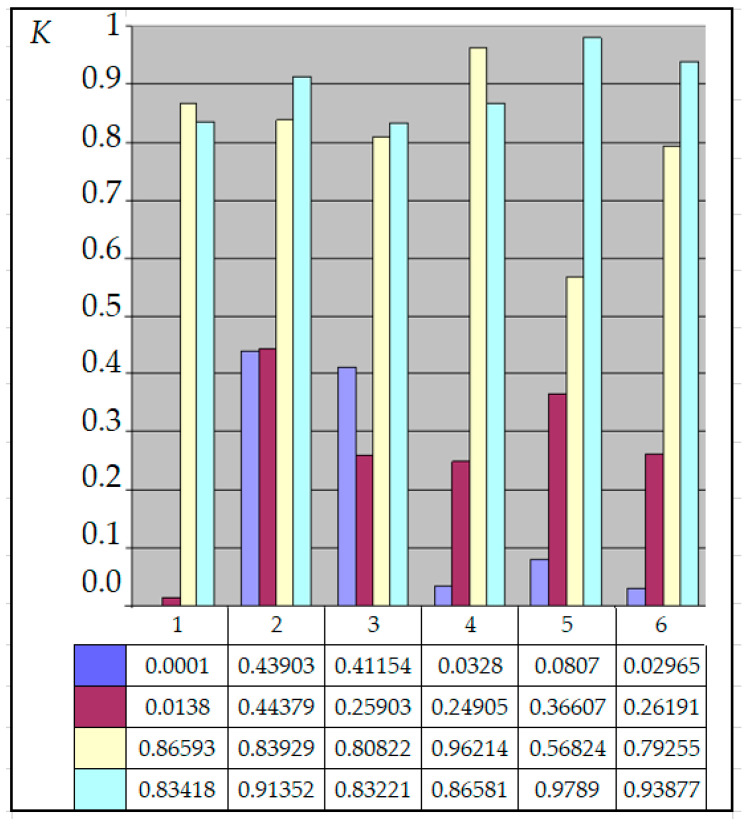
Estimation of the relationship between the tangent of the left branch slope of the fatigue curve and the number of stress cycles corresponding to the fatigue curve breaking point in the high-cycle area.

For the current study, the following samples of experimental results were considered: all data (1179 points); data on steels 30 (34 points), steels 40 (24 points), and steels 45 (115 points) (due to the prevalence of these steels (30, 40, and 45) in mechanical engineering and the large quantity of experimental data on fatigue for samples and parts made of these materials being present in the database); and data on the fatigue of copper and titanium (the latter structural materials were represented in the database by a few test results, 5 and 8 points, respectively). Each point under consideration represented the results of determining the fatigue curve equation parameters. Three fatigue parameters were compared in pairs: the fatigue limit, the abscissa of the fatigue curve breaking point (the number of stress cycles corresponding to the fatigue curve breaking point in the logarithmic scale), and the angle of inclination of the tangent of the fatigue curve to the axis of the number of cycles. The results are presented in Figure 5, Figure 6 and Figure 7. The selection of the best form for normalized fatigue parameters was made based on a comparison of the approximation confidence factor (K) when compared with each other in pairs. The coefficient of approximation reliability (the approximation confidence factor or coefficient of determination) (K) is a measure of how well the selected model fits in terms of explaining the variation in the selected variable. As an illustration of this procedure, Figure 5, Figure 6 and Figure 7 show the results of calculating the approximation confidence factors (K) when sequentially comparing the fatigue limit and the abscissa of the fatigue curve breaking point in the high-cycle region (Figure 5), the fatigue limit and the angle of inclination of the tangent of the fatigue curve’s left branch to the number of stress cycles axis for representing the fatigue curve in a logarithmic scale (Figure 6), and, finally, the abscissa of the fatigue curve breaking point in the high-cycle region and the angle of inclination of the tangent of the left branch of the fatigue curve to the number of stress cycles axis for fatigue curve representation in a logarithmic scale (Figure 7). 

The following combinations were considered:

Line 1—Initial values of fatigue parameters without any conversion;
Line 2—Logarithms of fatigue parameter values;
Line 3—Normalized values of fatigue parameters;
Line 4—Logarithms of normalized values of fatigue parameters.
Column 1—All data (1179 points);
Column 2—Data on steels 30 (34 points);
Column 3—Data on steels 40 (24 points);
Column 4—Data on steels 45 (115 points);
Column 5—Data on fatigue of copper (5 points);
Column 6—Data on fatigue of titanium (8 points).

Calculation results of the approximation confidence factor (K) for the indicated groups of fatigue curves for each of the four forms of fatigue parameter expression show that, for almost all options of calculating approximation dependencies, the maximum value of the approximation confidence factor was obtained for the normalized (recalculated) fatigue parameters proposed in the work.

The calculation results allow us to conclude that normalization increases the correlation confidence factor between fatigue parameters, regardless of the pair of compared fatigue parameters and the data sample. However, it should also be noted that the “gain” from applying the normalization procedure for different samples of experimental results is different.

In addition to calculating correlation coefficients, the relationship between data sets was assessed by calculating conditional entropy for the considered pairs of data sets (fatigue resistance indicators). To simplify the procedure for calculating entropy, the method of calculating cross-entropy using the box-counting method was used [15]. The value of cross-entropy is, in this case, a criterion for the degree of connection between two data sets, or a criterion for the predictability of values of one random variable provided by knowledge of another random variable in some way related to the first one, even if it is unknown in the form of analytical expression. The cross-entropy value was calculated in accordance with Equation (7):

(7) $$I(X,Y)=\log\frac{N_{x}N_{y}}{N_{xy}},$$

where *N_xy_* is the total number of cells in the merged space; *N_x_* is the number of cell projections onto space *X*; $\frac{N_{xy}}{N_{x}}$ is the characteristic scatter of points along the *Y* axis at a fixed *X*; and *N_y_* is the characteristic spread of all data along the *Y* axis.

The cross-entropy value provides an estimate of the logarithm of a typical spread of values of variable Y as a ratio of the typical spread of this variable, but with a known variable *X* value. The greater the cross-entropy, the more certainty is brought by the knowledge of the *X* value to the prediction of the variable *Y* value. In the current case (when it was necessary to solve the problem of selecting a form expressing the relationship between the fatigue resistance parameters of metals), the calculation of cross-entropy made it possible to choose such options for converting experimental data, in which the scatter of data relative to the approximated dependence was minimal [26,30,31]. 

Box-counting algorithms are based on counting the numbers filling the considered set of cell points (boxes) with values, into which the space formed by the values of the argument and function, is specially divided. 

Occupancy numbers are used to estimate the probable density of example distribution across the cells. A set of probabilities obtained in this way allows one to make an assessment of any statistical characteristic of the sample under consideration. The employed entropy analysis does not require any assumptions about the nature of the relationship between input and output variables. Thus, it provides the most general way to determine the significance of the original data presentation form (input), allowing one to assess the resulting variable degree of predictability—i.e., the desired function (output).

**Figure 8:**
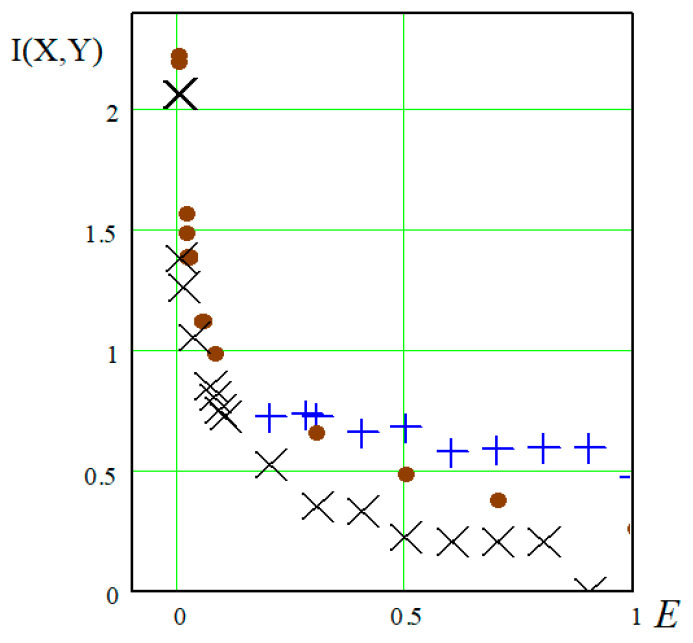
Results of cross-entropy calculation using the box-counting method: “+”—the combined space is formed by fatigue parameters in the original form (1); “×”—the combined space is formed by partially transformed fatigue parameters (2); “•”—the combined space is formed by the recalculated fatigue parameters (3).

Figure 8 shows the results of calculating cross-entropy for various combinations of fatigue parameters: in case of using them in the original form (1), in case of various options for normalizing fatigue parameters (2)—as one of the subsequently excluded options, and (3) as the best option for transforming the experimental results [26].

The abscissa axis shows the sizes of the cells into which the combined space was divided (in fact, this is the magnitude of the error in determining the required parameters). Cross-entropy calculations based on experimental data represented by the coordinates of points on the plane were carried out using the developed Box-Counting program [26].

The method of calculating cross-entropy was used as an alternative method, independent of the previous one (with assessment of the approximation confidence factor), to identify the preferred form for expressing the recalculated fatigue parameters for comparing mix as part of a generalized dependence. Therefore, it is not possible to judge the equality K = I(x,y), but in meaning and in terms of the purpose of their use, these are similar “tools”—both methods allow us to choose the best form among those considered for the recalculated fatigue parameters.

The performed correlation and covariance analysis of data allowed us to select, as the best, the following form for the recalculated fatigue parameters (Equations (8)–(10)):

σ_re_ = −log(σ*_R_*/σ_*_), (8)

*N*_re_ = −log(*N_G_*/N_*_), (9)

tgα*_W_*_re_ = −log(tgα*_W_*). (10)

**Figure 9:**
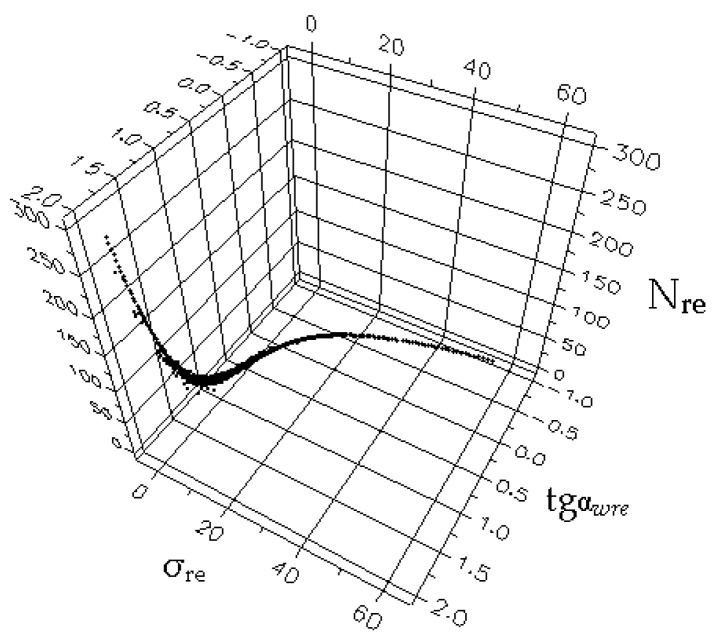
Generalized dependence of the recalculated fatigue parameters with the scales of the change of these values.

The generalized dependence of the recalculated parameters was obtained by a joint consideration of over a thousand fatigue test results conducted by various authors to determine the breaking point coordinates of fatigue curves in the high-cycle fatigue region, as published in the literature. In Figure 9 [9], the dependence of the recalculated fatigue parameters is shown schematically. 

**Figure 10:**
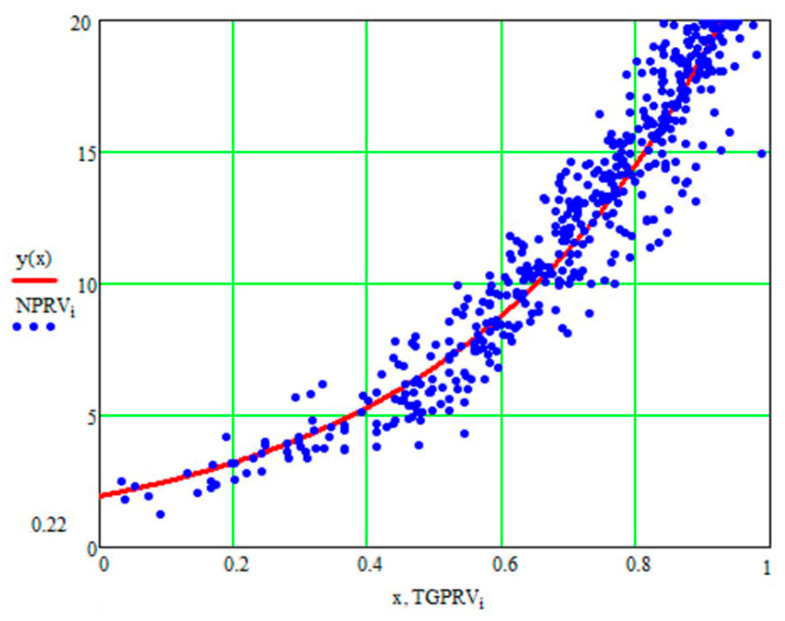
Comparison of the recalculated fatigue life and the recalculated fatigue curve angle of inclination to the number of stress cycles axis (recalculated fatigue life—NPRV; recalculated inclination angle—TGPRV); y(x)—approximation function (Formula (11)).

**Figure 11:**
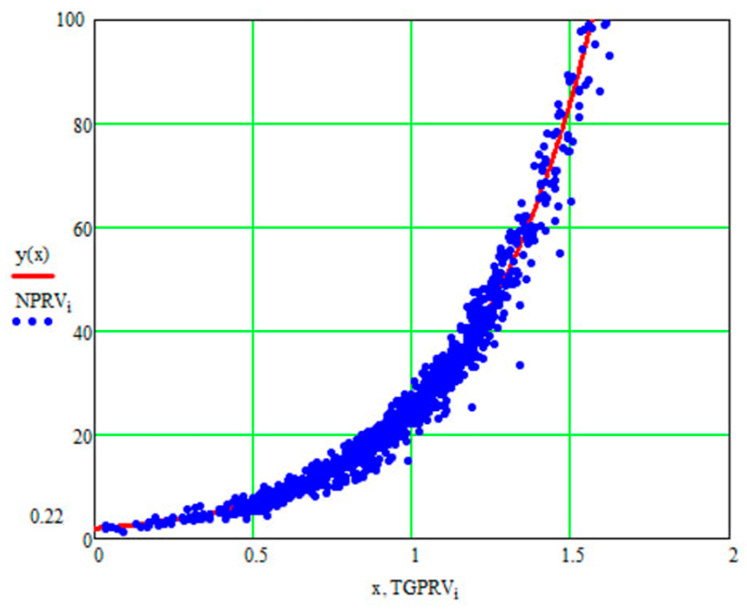
Comparison of the recalculated fatigue life and the recalculated fatigue curve angle of inclination to the number of stress cycles axis (recalculated fatigue life—NPRV; recalculated inclination angle—TGPRV). Compared to Figure 9, the scale along the abscissa and ordinate axis has been changed; y(x)—approximation function (Formula (11)).

**Figure 12:**
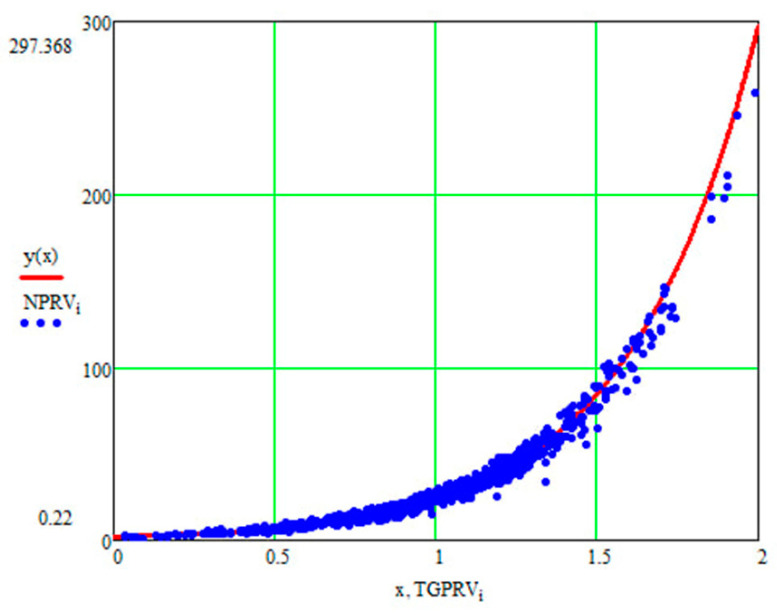
Comparison of the recalculated fatigue life and the recalculated fatigue curve angle of inclination to the number of stress cycles axis (recalculated fatigue life—NPRV; recalculated inclination angle—TGPRV); y(x)—approximation function (Formula (11)) Compared to Figure 10, the scale along the ordinate axis has been changed.

Figure 10, Figure 11 and Figure 12 show one of the projections of this generalized dependence onto the coordinate planes of the recalculated space of fatigue parameters. This is the dependence of the recalculated fatigue life and the recalculated fatigue curve angle of inclination to the number of stress cycles axis. The different scales of the coordinate axes in these figures make it possible to confirm the universal nature of the generalized dependence and the degree of accuracy in selecting the parameters of the approximating expression.

Analytical expressions for the generalized dependence (Equations (11)–(13))

N_re_ = 1.9421exp(2.5156 tgα*_W_*_re_), (11)

σ_re_ = 6.2565exp(−2.2945 tgα*_W_*_re_), (12)

σ_re_ = 11.028 N_re_^−0.8997^. (13)

There are two main results of the studies performed. The first is the obtained method of transforming experimental information, which allows one to compare the transformed parameters of high-cycle fatigue with each other—the fatigue limit, the abscissa of the fatigue curve breaking point in the high-cycle region, and the structurally sensitive parameter of fatigue—the slope of the left branch of the fatigue curve in the high-cycle region to the number of stress cycles axis. This method allows, within the single-dependency structure, to jointly compare experimental information on fatigue obtained with respect to a large number of combinations of operating factors.

In this transformed space, information about the fatigue limits considered in the study of fatigue curves allows one to obtain almost functional relationship between fatigue parameters, which can be used as the basis for various methods of predicting fatigue parameters and fatigue curves. 

The second result is related to the first one—the explicit form of the generalized dependence of normalized fatigue parameters allows us to quantify the nature of the relationship between the fatigue parameters. While only a qualitative level of the tendency to connect the fatigue limits and behavior of the left steeply inclined branch of the fatigue curve (the steeper the left section of the fatigue curve is inclined in the high-cycle region, the lower the fatigue limit) is mentioned in the literature, with a generalized dependence of the normalized fatigue parameters it is possible to quantify the relationship between three fatigue parameters in the high-cycle region (the fatigue limit, the abscissa of the fatigue curve breaking point in the high-cycle region, and the structurally sensitive parameter of fatigue—the tangent of the left branch slope of the fatigue curve in the high-cycle region to the number of stress cycles axis).

Thus, this study made it possible to identify a practically functional relationship between high-cycle fatigue parameters. If earlier it was possible only imply the observed trend in the joint change of two high-cycle fatigue parameters of resistance (steeper or lower), now it is possible to view this connection explicitly. It is also possible to define the parameters of this connection.

Another result is also based on the connection between the fatigue limit coordinates of the fatigue curve in the high-cycle region and the structurally sensitive parameter of fatigue resistance, which allows us to view this as a parameter that integrally describes the process of damage accumulation during loading at stress levels exceeding the fatigue limit. Additionally, the fatigue curve behavior at stress levels exceeding the fatigue limit determines the fatigue limit position. The latter provision makes it possible to develop various methods for the accelerated construction of fatigue curves in the multi-cycle region, based on limited-volume fatigue tests undertaken to estimate the angle of inclination of the tangent of the fatigue curve’s left branch to the number of stress cycles axis. These methods and the generalized dependence of the recalculated fatigue parameters will make it possible to predict the fatigue curve in the high-cycle region.

## 4. Discussion

The design of experimental results that form a generalized dependence on coordinate planes formed by the coordinate axes of recalculated strength, recalculated fatigue life, and recalculated fatigue curve angle of inclination to the number of stress cycles axis made it possible to obtain analytical expressions for the dependences of recalculated parameters.

The recalculated fatigue parameter expression includes the parameters that determine the fatigue curve breaking point in the high-cycle region—the fatigue limit and the abscissa of the fatigue curve breaking point. These values were experimentally obtained, rather than geometrically processing the fatigue curve rectified in a logarithmic scale. This was performed in contrast to obtaining conventional point coordinates at which the extended left branch of the fatigue curve intersects the abscissa and ordinate axis. In this sense, the recalculated strength and recalculated fatigue life contain values obtained independently of the tangent of the fatigue curve slope in the high-cycle region. However, in the sense of the physical process of damage accumulation and the destruction of a laboratory sample or detail under cyclic loading, the identified generalized dependence confirms the existence of a connection between the tangent of the left branch slope of the fatigue curve in the high-cycle region and the fatigue limit coordinates, both in terms of stress value and the number of cycles corresponding to the fatigue curve breaking point. This is in good agreement with the general idea that the combination of acting factors determines the damage accumulation process rate under cyclic loading, the duration of characteristic stages in the process of a laboratory sample or detail destruction, and, consequently, a certain fatigue curve angle of inclination in the high-cycle region. The generalized dependence of the normalized fatigue parameters also provides an addition to this construction in the sense that the fatigue limit, being also a consequence of a combination of factors, will be eventually associated with the angle of inclination of the tangent of the fatigue curve to the number of stress cycles axis.

The authors adhere to such a view on the nature of fatigue, according to which the test environment, along with other active factors (test conditions), produces a unique set of active factors, their joint action resulting in forming the process of damage accumulation in the structural material that is specific to the given experimental conditions. This will lead to a certain, unique result of the ratio of each damage accumulation stage duration corresponding to different stress levels and, accordingly, its value being equal to the number of stress cycles preceding the destruction of the sample. As a result, a unique value of the structurally sensitive indicator of fatigue resistance—the angle of inclination of the left branch of the fatigue curve in the high-cycle region to the axis of the number of cycles—and a unique value of the fatigue limit and the number of cycles to failure at the fatigue limit, which are fatigue curve breaking point coordinates in the high-cycle region, were obtained.

Obtaining a generalized dependence was preceded by the procedure of the fatigue curve information conversion, containing data on the fatigue limit, the number of stress cycles (the number of cycles to failure at the fatigue limit or, in other words, the abscissa of the fatigue curve breaking point in the high-cycle region), a structurally sensitive indicator—the angle of inclination of the tangent of the fatigue curve to the number of stress cycles axis—for representing a fatigue curve in a logarithmic scale. The calculation of recalculated fatigue parameters allows information about a specific fatigue curve to be converted into three indicators called recalculated fatigue parameters, which can be thought of as the point coordinates in three-dimensional space of the recalculated fatigue parameters. Repeating this reduction procedure allows us to translate the considered fatigue curve into the recalculated fatigue parameters space. And so on. Joint consideration in the recalculated space of points corresponding to the considered fatigue curves implies obtaining a generalized dependence. This dependence, resulting from the joint processing of a large number of fatigue curves obtained from a wide range of sets of acting factors, can be considered a universal dependence. The transformation performed enables the transfer of fatigue curves (i.e., information about their parameters) from a traditional logarithmic coordinate system into the recalculated parameters space, in which the parameters previously not showing a tendency to simple functional comparison now clearly demonstrate a functional relationship that can be used to implement forecasting methods—i.e., accelerated methods for fatigue curve prediction.

Analysis of such comparison results between operating factors and fatigue indicators shows that a certain set of operating factors determines the value of structurally sensitive parameters of fatigue resistance—the angle of inclination of the tangent of the fatigue curve to the number of stress cycles axis—for straightening the fatigue curve in a logarithmic scale, and this parameter value will determine the position of the fatigue limit. Hence, the proposed generalization method is based on joint consideration of fatigue limits and structure-sensitive fatigue resistance parameters for a large number of fatigue curves, making it possible to explicitly identify a generalized dependence in the normalized space of recalculated parameters. The transformed information about a particular fatigue curve was mapped into the recalculated fatigue space and it was compared with similarly transformed information of a large number of other fatigue curves. The basis for this transformation is formed by the comparison of the fatigue curves of metals and alloys with data on fatigue curves for which fatigue limits were obtained during the experiments (breaking points on the fatigue curve in the high-cycle region for straightening in the logarithmic scale). A large number of fatigue curves are considered simultaneously. They were obtained under a wide range of varying experimental conditions and for a wide range of numerical parameters determining the current experimental conditions.

Presenting all of the selected results in one figure (Figure 3) makes it possible to understand the existing difficulties in studying factors that influence the strength properties of a material under cyclic loading and, as a consequence, the position of fatigue curves. Indeed, the variety of possible combinations of acting factors leads to changes in the fatigue curves’ breaking point coordinates within too wide limits. One can see that the characteristics of this set of points without any recounts can only be of a semi-qualitative nature and can only approximately describe the area occupied by the fatigue curves’ breaking points.

Figure 8 and Figure 12, showing the generalized dependence of the recalculated fatigue parameters, and Figure 2, showing the experimentally determined fatigue curves’ breaking points in the high-cycle region, present the same experimental results. It can be seen that the use of information about fatigue in a transformed form, in the form of recalculated fatigue parameters, provides an opportunity to develop various methods for predicting fatigue parameters and fatigue curves based on the generalization of experimental data. However, it is obvious that the transformation, on the one hand, helps to generalize information and makes it possible to obtain a functional relationship between the recalculated fatigue parameters, on the other hand, it complicates the intuitive perception of how fatigue curves, traditionally presented in a logarithmic scale, will be displayed in recalculated space of fatigue parameters. In order to illustrate the result of such fatigue curve mapping constructed on logarithmic scales in the space of recalculated fatigue parameters, we considered the special cases of mapping certain conditional fatigue curves in which out of three fatigue parameters (fatigue limits, abscissa of fatigue curve breaking points in the high-cycle region, and the angle of inclination of the tangent of the left branch of the fatigue curve to the number of stress cycles axis [26]) two were fixed, with the last indicator varying within a certain range.

It was found that, with an increase in the angle of inclination of the tangent of the fatigue curve to the number of stress cycles axis, the fatigue curve, being steeper, moves into the region of lower fatigue limit values, and, vice versa: with a decrease in the fatigue curve inclination, the latter will be located more flatly, moving into the region with higher fatigue limit values. To quantitatively confirm this observation, a fatigue curve was selected with some “average” values of the fatigue curve breaking point coordinates in the high-cycle region and the angle of inclination of the fatigue curve to the number of stress cycles axis. Then, this fatigue curve position was calculated onto the generalized dependence of the recalculated fatigue parameters, as was its displacement relative to its original position when changing coordinates according to a certain rule. For this purpose, three typical situations were considered:

- At a constant angle of inclination of the fatigue curve to the number of stress cycles axis and a constant fatigue limit value, the abscissa (number of stress cycles) of the fatigue curve breaking point changed;
- At a constant angle of inclination of the fatigue curve to the number of stress cycles axis and a constant abscissa of the fatigue curve breaking point, the fatigue limit value changed;
- At constant fatigue limit values and the abscissa of the breaking point, the angle of inclination of the fatigue curve to the number of stress cycles axis changed.

**Figure 13:**
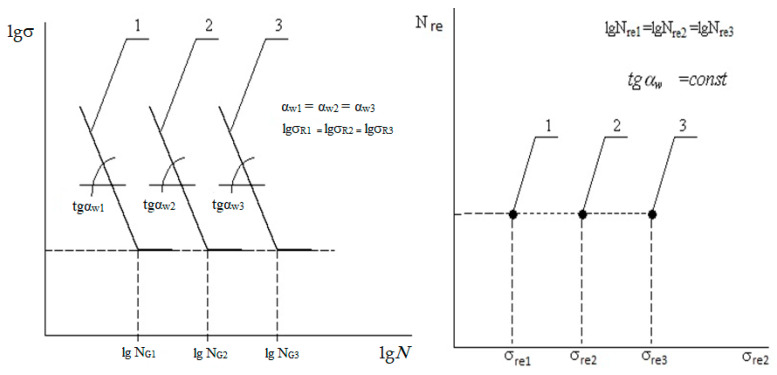
A scheme mapping the high-cycle region of three fatigue curves with the same fatigue limit value into the space of recalculated fatigue parameters.

An increase in the abscissa of the fatigue curve breaking point, all other things being equal (that is, without changing the fatigue limit and the angle of inclination of the left branch of the fatigue curve to the number of stress cycles axis) leads to a movement in the space of the recalculated point indicators corresponding to the fatigue curve. This movement occurs along a straight line, which is the intersection of two planes in which the values of *N*_re_ and tgα*_W_*_re_ do not change [7,26]. In this case, an increase in the abscissa of the fatigue curve breaking point causes an increase in the recalculated strength (σ_re_), and its decrease leads to a decrease in σ_re_. Consequently, if two fatigue curves with the same slope angles and the same fatigue limit value are considered, the curve with a larger abscissa value of the fatigue curve breaking point will have a larger σ_re_ value. This is shown in Figure 13.

Changing the fatigue limit value, all other things being equal (that is, without changing the abscissa of the fatigue curve breaking point and the angle of inclination of the fatigue curve to the number of stress cycles axis) causes the fatigue curve movement (a point in the space of the recalculated fatigue coordinates) along a straight line, which is the intersection of two planes, in which σ_re_ and tgα*_W_*_re_ do not change their values. In this case, an increase in the fatigue limit results in a recalculated fatigue life increase and a decrease in the fatigue limit, accordingly, leads to a decrease in the recalculated strength.

**Figure 14:**
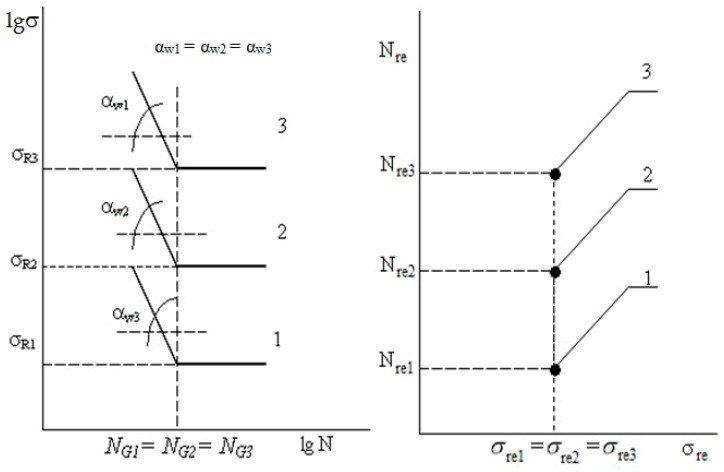
A scheme mapping the multi-cycle region of three fatigue curves with identical angles of inclination to the axis of the number of cycles and abscissas of breaking points into the space of recalculated fatigue parameters.

Thus, if two fatigue curves with the same slope angles and the same values of the abscissa of the breaking point are considered, the curve with the larger fatigue limit value will have a greater value of its recalculated fatigue life. This is shown in Figure 14.

**Figure 15:**
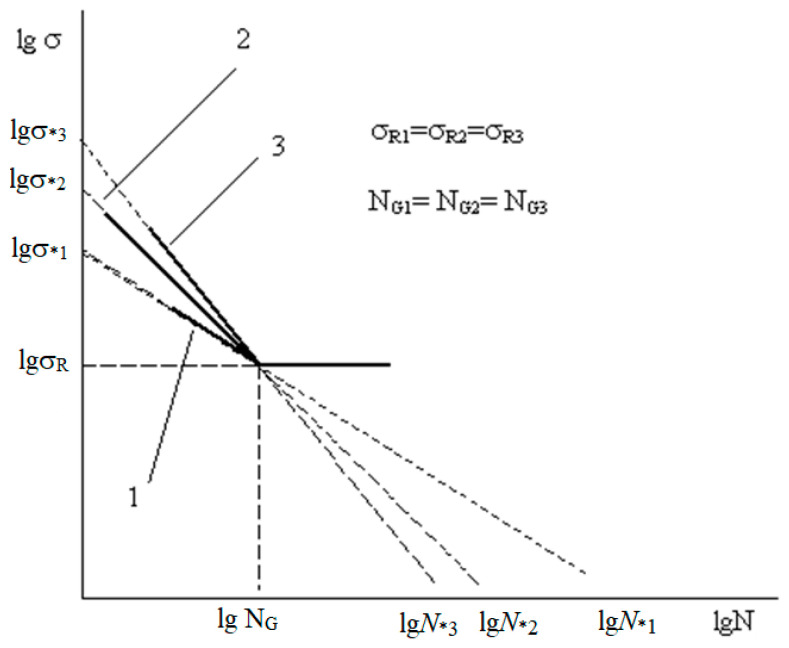
Schematic representation of the high-cycle region of three fatigue curves with the same abscissas of breaking points and values of fatigue limits, but different values of angles of inclination to the number of stress cycles axis.

Comparing fatigue curves by inclination angle in a similar formulation of the problem, as in the two previous cases, is impossible since changing the inclination angle up or down leads to a more complex and ambiguous relationship between σ_re_ and *N*_re_. Let us consider three fatigue curves for which the angle of inclination varies within certain limits, and the breaking point coordinates for all three curves in the multi-cycle region coincide (Figure 15). The peculiarities of the procedure for constructing the space of recalculated fatigue resistance parameters allow us to conclude that such a situation is a case of the inaccurate determination of fatigue curve breaking point coordinates. Indeed, a certain set of operating factors corresponds to a certain structure-sensitive parameter value of fatigue resistance—the angle of inclination of the fatigue curve to the number of stress cycles axis. Each inclination angle value corresponds to a certain value of fatigue curve breaking point coordinates. Therefore, it is possible to talk about an interrelated change in the recalculated fatigue indicators, reflecting the difference in values of the structure-sensitive parameter for the compared fatigue curves.

Calculation of the recalculated fatigue parameters and consideration of the fatigue curves’ position relative to the generalized dependence of the recalculated fatigue parameters in this case produces the following scenario: with an increase in the angle of inclination of the tangent of the fatigue curve to the axis of the number of cycles in the high-cycle region, tgα*_W_*_re_ decreases. At the same time, the recalculated fatigue life N_re_ decreases while σ_re_ increases. Thus, in the generalized dependence, the region with the maximum N_re_ value corresponds to a group of fatigue curves with a small angle of inclination to the axis of the number of cycles and they are characterized by small σ_re_ values. On the contrary, the region with large inclination angles is characterized by small N_re_ values and large σ_re_ values. Taking some characteristic values of the angle of inclination of the fatigue curve to the axis of the number of stress cycles (for example, the maximum, intermediate, and minimum), three characteristic ranges of values for the recalculated fatigue parameters were obtained. For these conventionally selected areas, the following relationship between fatigue parameters can be written in the traditional form and in the form of recalculated fatigue parameters. For fatigue curves with relatively small angles of inclination to the axis, the numbers of recalculated stress cycles N_re_ and tgα*_W_*_re_ have maximum values, and σ_re_ has minimum values. Conversely, for fatigue curves with relatively large angles of inclination to the axis, the numbers of stress cycles N_re_ and tgα*_W_*_re_ have minimum values, and σ_re_ has maximum values.

In these characteristic areas, depicting the displacement direction of points which reflect the fatigue curve’s position in the space of recalculated fatigue parameters, taking place when the breaking point position in the logarithmic scale changes, a diagram that makes it possible to compare the points corresponding to the experimental fatigue curves in the space of recalculated fatigue parameters was obtained. This diagram, showing generalized dependence characteristic areas of the recalculated fatigue parameters and the relationships between them, allows one to compare the fatigue curves of machine parts and structures with simultaneous consideration of all three characteristics of the fatigue curve position in the high-cycle area (tgα*_W_*_re_, N_G_, and σw_re_). It also allows one to interpret the results of displaying fatigue curves into the space of recalculated fatigue parameters more accurately and to prepare initial data for obtaining an analytical expression of factor adjustment functions, taking into account the impact of existing factors.

**Figure 16:**
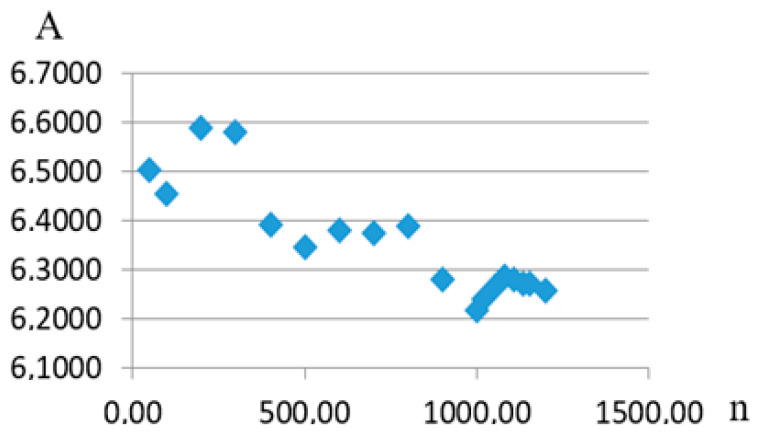
Dependence of coefficient A on the number of fatigue curves considered (n).

**Figure 17:**
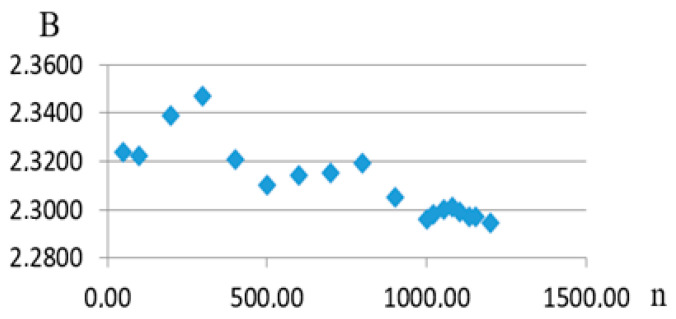
Dependence of coefficient B on the number of fatigue curves considered (n).

The results of the study of the saturation process determining the generalized dependence parameters can be cited in order to illustrate this. It should be noted that the authors are handling 1179 fatigue curves. Considering the expression for one of the generalized dependence projections onto the plane formed by coordinate axes of the recalculated strength and the recalculated angle of inclination of the fatigue curve to the axis of the number of stress cycles, an approximating expression was obtained (9). Writing it as σ_re_ = Aexp(−Btgα*_W_*_re_), it is possible to show how the A and B coefficients’ values change as the number of fatigue curves considered as part of the generalized dependence increases (Figure 16 and Figure 17).

It is noteworthy that the values used to obtain the recalculated fatigue parameters, on the one hand, were obtained experimentally (these are the fatigue curve breaking point coordinates in the high-cycle region—the fatigue limit and the abscissa of the fatigue curve breaking point in the fatigue curve high-cycle region), and, on the other hand, conditional ones were also used in physically unrealizable quantities—the value of the stress logarithm and the number of cycles logarithm at which the left branch of the fatigue curve, steeply inclined in the fatigue curve high-cycle region, intersects the axes of logarithmic coordinates. There is a possibility of considering such a procedure—relating each current value of stress and the number of cycles, taken as the points’ coordinates belonging to the fatigue curve, to the values of conditional, physically unrealizable values of stress and the number of cycles, in which the conditionally extended left branch of the fatigue curve intersects the coordinate axes. The fatigue curve is normalized in a recalculated coordinate system.

All fatigue curves, regardless of the specific combination of fatigue parameters (fatigue limit, angle of inclination of the tangent of the left branch of the fatigue curve to the axis of the number of stress cycles and fatigue life, corresponding to the breaking point) are transformed in the recalculated coordinate system into exponential curves—these are the generators (L) of certain conditional surfaces. Next, an additional coordinate axis was considered, on which the tangent of the fatigue curve will be plotted, assuming that other fatigue indicators are associated with this parameter. The guide forming this surface, for example, m, will be a straight line parallel to the axis of change of the tangent of the left branch of the fatigue curve, along which the generator L will shift. Now, let us mark the position of the fatigue curve breaking point in the high-cycle region on each such normalized fatigue curve and consider these points together.

Thus, the above generalized dependence of recalculated fatigue parameters is obtained. Thus, the dependence that could only be suspected before, based on weak correlations revealed between, for example, the tangent of the slope of the fatigue curve’s left branch and the fatigue life and limit in the high-cycle region, after such a transformation takes on an almost functional form. Obviously, such dependence parameters are very sensitive to the accuracy of determining the fatigue curve breaking point coordinates in the high-cycle region, but a sufficiently large number of fatigue curves considered together (1179 fatigue curves are part of the generalized dependence) makes it possible to identify the generalized dependence empirically, without forming any physical, chemical, or mathematical justification. In this case, it is possible to say that the generalized dependence is obtained based on the provisions of generalized analysis. Initially, when transforming coordinate systems to present information about fatigue tests, it is possible to obtain dependences that are practically useful for forecasting. Their calculation results can be further used and visualized in traditional coordinate systems.

**Figure 18:**
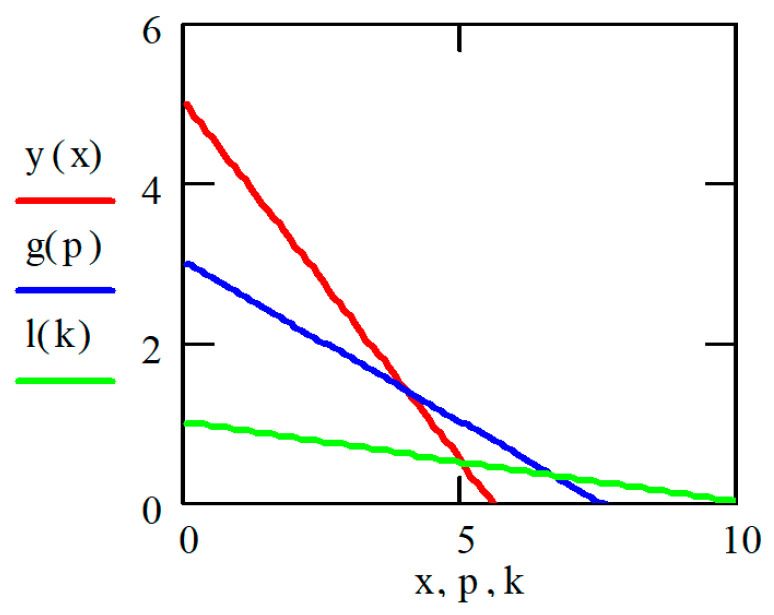
Three arbitrary straight lines, similar to the fatigue curves conditionally extended to the intersection with the coordinate axes, rectified in the high-cycle region (n).

**Figure 19:**
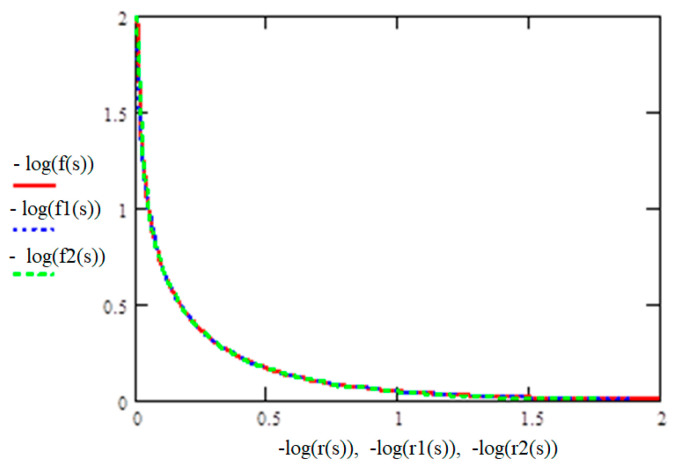
The same lines after normalization, which consists of dividing each point corresponding to coordinates belonging to the line under consideration by the ordinate or abscissa values of the points at which the line in question intersects the coordinate axis and the subsequent logarithm of the resulting values to obtain the point abscissa and ordinate it in the recalculated space (that is, the transformation of traditional coordinates of the lines under consideration into a reduced (normalized) space).

Below is an example of applying a normalization procedure that transfers three arbitrary straight lines (Figure 18) to a surface, the generators of which are shown in Figure 19.

**Figure 20:**
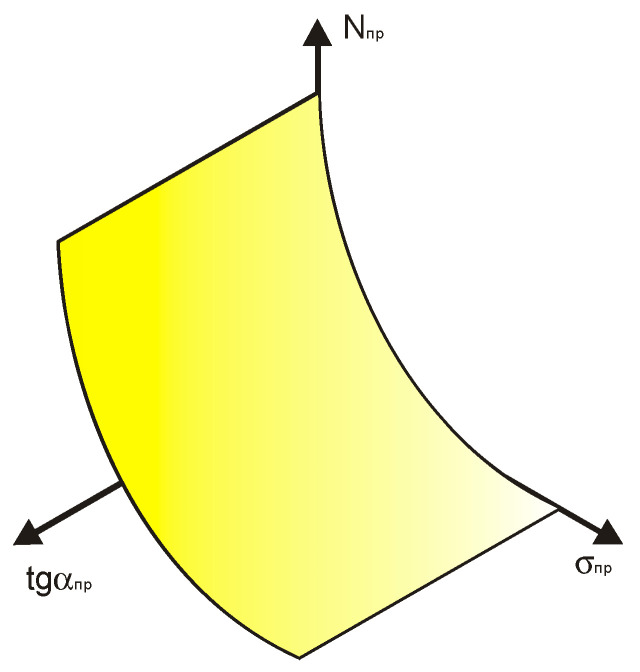
Schematic representation of the surface within which the normalized fatigue curves are compared with the angle of inclination of the tangent to the axis of the number of stress cycles.

A schematic representation of the surface under consideration is presented in Figure 20 [9].

**Figure 21:**
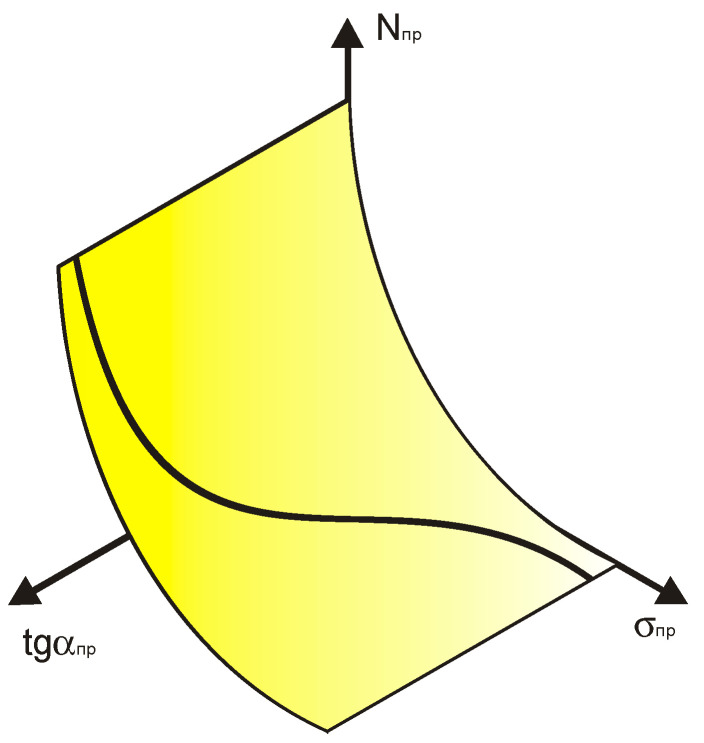
Schematic representation of the recalculated fatigue parameter surface with the points highlighted corresponding to the fatigue curve breaking points of metals and alloys associated with the fatigue curve slope in the high-cycle region.

The next stage of the transformation is to jointly consider all the compared fatigue curves’ breaking points in the recalculated coordinate system. These data represent the fatigue experiment results obtained by different authors, who carried them out in order to determine the physical fatigue limit position in the high-cycle part of fatigue curve (Figure 21 [9]).

**Figure 22:**
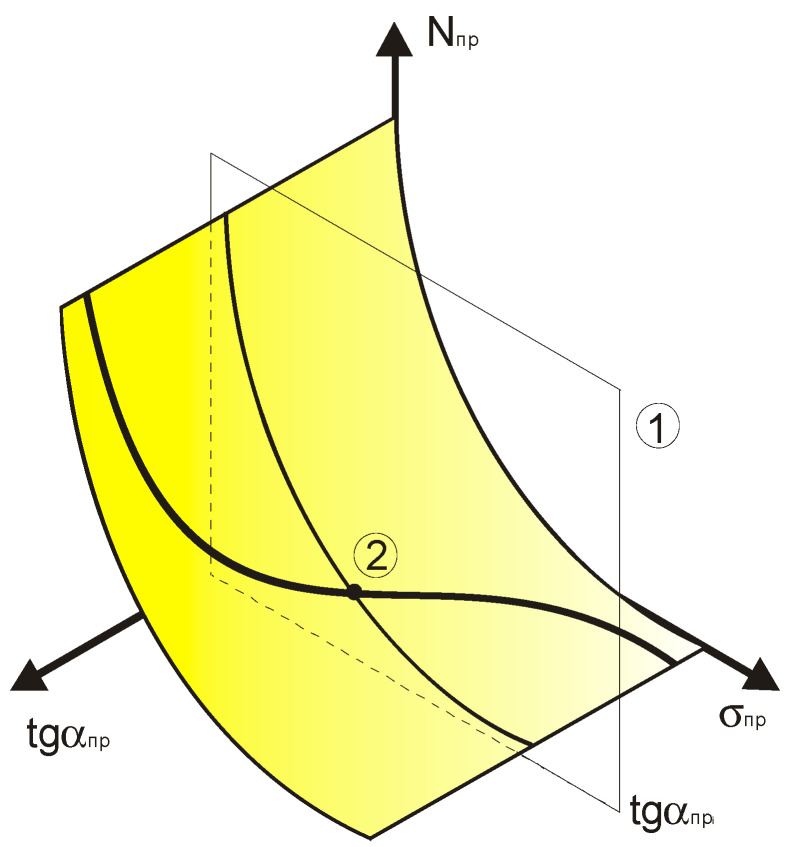
Simplified diagram of the method for predicting high-cycle fatigue parameters for metals. (1) A plane corresponding to the angle of inclination of the fatigue curve to the axis of the number of stress cycles found by calculation or experiment; (2) the result of determining the fatigue limit position on the generalized dependence of the recalculated fatigue parameters.

The resulting generalized dependence of normalized (recalculated) fatigue parameters can be used as part of a method for the accelerated construction of fatigue curves, which solves the same problems as methods for accelerated construction of fatigue curves discussed in [1,7,8,32,33,34,35,36,37]. By choosing relatively high stress levels and performing fatigue tests on several samples, it is possible to determine the angle of inclination of the tangent of the fatigue curve’s left branch to the axis of the number of stress cycles. This parameter allows to determine the point coordinates on the generalized dependence corresponding to this value of the slope angle of the fatigue curve’s left branch. Figure 22 shows a diagram explaining the procedure for determining the point on the generalized dependence corresponding to the experimentally obtained slope angle of the fatigue curve’s left branch.

By restoring the fatigue limit value and the abscissa of the fatigue curve breaking point in the high-cycle region from the recalculated values, it is possible to complete construction of the predicted fatigue curve. To verify the obtained prediction, it is necessary to conduct another control test at stress levels not exceeding the predicted fatigue limit value. Thus, instead of testing 10–15 samples to construct a fatigue curve in the high-cycle region, it is possible to test a smaller number of samples at higher stress levels, which reduces the test duration [37].

Performing fatigue verification tests on laboratory samples, as well as the fatigue testing of a number of full-scale parts, for example, piston pins or steering linkage arms, made it possible to evaluate the method’s margain of error in predicting the fatigue limit and enabling accelerated construction of the fatigue curve in the high-cycle region. The margain of error in determining the fatigue limit was within the 13.8% to 3.9% range and the error in determining the abscissa of the fatigue curve breaking point was within the 25.8% to 4.2% range [37].

Verification of the method for predicting the fatigue curve and the generalized dependence parameters should be carried out employing data that was not used in developing the prediction method. The information (benchmark) as a source of such data for verification was collected in the FatLim database [38,39,40].

**Figure 23:**
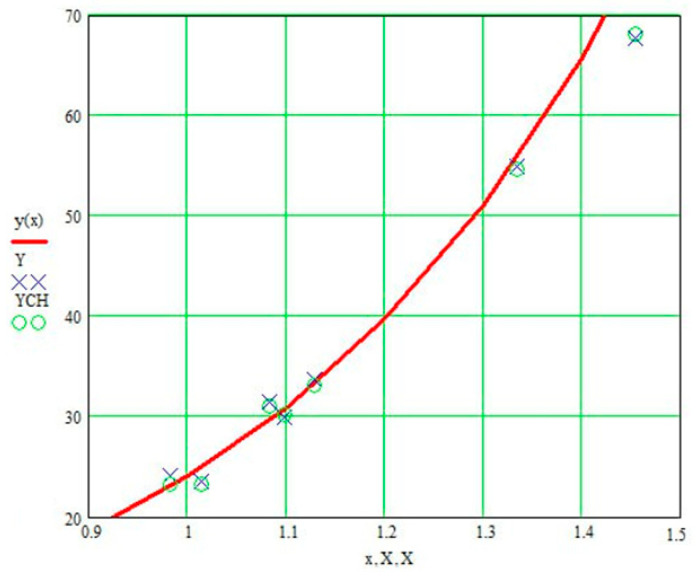
Recalculated fatigue life dependence on the recalculated angle of inclination. The red line is a generalized dependence section, the green dots are the results of predicting the fatigue limit using the FatLim database, the blue crosses are the results of predicting the fatigue limit using the developed method.

This makes it possible to independently check the parameters of the generalized dependence. For example, seven fatigue curves, designated in the FatLim database as BaB09, BaB06, BaB04, BaB02, Bai14, Bai01, and Bai02, were randomly selected for testing. The comparison results are shown below. In Figure 23, the solid line marks the section of the generalized dependence of the recalculated (normalized) fatigue parameters, the green dots show the results of predicting the fatigue limit using the FatLim database, and the blue crosses show the results of predicting the fatigue limit using the proposed method. These values are presented in the space of recalculated fatigue parameters. According to the figures, the fatigue parameters declared in FatLim and calculated by the proposed method of accelerated fatigue curve construction practically coincided in the space of recalculated fatigue parameters for all selected fatigue curves.

The main verification result obtained is as follows. The indicated fatigue curves of samples made of CK35 steel consisting of a pipe of different internal diameters and total lengths, under tensile compression loading with a constant harmonic amplitude, demonstrate good agreement with the results of assessing the generalized dependence parameters of the recalculated fatigue parameters. The authors realize that the observed deviation of the recalculated fatigue parameters’ generalized relationship is associated with a certain set of empirical information about the high-cycle fatigue experiments that were used to estimate the generalized relationship parameters. If the database is replenished with new data, the generalized dependence parameters will be refined, and the more data is taken into account, the more reliable the parameters of the generalized dependence will be.

## 5. Conclusions

Within the framework of the current article:

1. Experimental information accumulated to date on the fatigue of metals and alloys was summarized and systematized.

Experimental data on 1179 fatigue curves of metals and alloys, obtained by a large number of authors and published in the literature, were systematized in the current work. The range of changes in fatigue limits for the considered fatigue curves is from 10 MPa to 1050 MPa. Abscissas of the breaking points of the considered fatigue curves range from 2 × 10^4^ to 8 × 10^8^ stress cycles. The angle of inclination of the tangents of the fatigue curves to the axes of the number of stress cycles for representing the fatigue curve in a logarithmic scale vary in the range from 0.01 to 7.692. Such a wide range of changes in fatigue parameters was achieved by widely varying combinations of operating factors—test conditions and large ranges of changes in parameters that quantitatively characterize the operating factors. The fatigue curves dealt with in the study were obtained for 204 different grades of steels and alloys; tested under 17 different loading schemes; under 54 test environments; 66 different test temperatures; for 4 different cross-sectional shapes of samples or parts; with 63 different values of the ratio of the surface area of the sample to its volume (for characterizing the scale factor); with 65 different values of characteristic cross-sectional size of the sample; 68 different values of operating load frequency; 81 thermal processing modes; 28 different surface treatment methods; and 63 different degrees of surface finish.

2. The collected data were normalized and the generalized dependencies were obtained.

Joint processing of the systematized fatigue curves using the procedure proposed by the authors to normalize the fatigue parameters to their recalculated analogues made it possible to obtain a generalized dependence of the recalculated (normalized) fatigue parameters and to evaluate its form. The parameters’ sensitivity analysis was performed that determined the form of the generalized dependence and the type of expression for the normalized (recalculated) fatigue parameters. This analysis was based on the results of calculating the degree of connection between data sets, representing different options for expressing the recalculated fatigue parameters.

3. A method for predicting fatigue indicators based on this generalized relationship was developed and its accuracy was evaluated by comparing it to the experimental results of fatigue tests and other methods of predicting of high-cycle fatigue indicators.

Analysis of the generalized dependence of recalculated fatigue parameters confirmed the universal connection between the tangent of the left branch of the fatigue curve, the fatigue limit, and the fatigue life of the fatigue curve’s breaking point in the high-cycle region, which can be used for accelerated prediction of fatigue curves. Verification tests performed both for laboratory samples and for a number of full-scale parts made it possible to estimate the error in determining fatigue parameters. The error in determining the fatigue limit was within the 13.8% to 3.9% range, and the error in determining the fatigue life for the fatigue curve breaking point was within the 25.8% to 4.2% range.

## Data Availability

Data are contained within the article.

## Nomenclature

lg*N*	logarithm of the stress cycle number;
σ	the stress, MPa;
lgσ*_R_*	logarithm of the stress corresponding to the fatigue limit;
lg*N_G_*	the abscissa of the fatigue curve breaking point (logarithm of the number of stress cycles corresponding to the fatigue limit and the fatigue curve transition to an almost horizontal section);
α*_W_*	the structure-sensitive parameter of metal fatigue in a logarithmic coordinate system;
tgα*_W_*	the tangent of the fatigue curve slope in the high-cycle region;
lgσ_*_, lg*N*_*_	the conditional, physically unrealizable values of the stress and number of cycles logarithms at which the straightened fatigue curve intersects the coordinate axes;
w	the frequency of the applied stress cycles when testing laboratory samples or parts for fatigue;
CF	the coefficient for the assessment of the influence of stress cycle frequency;
N_G_/N_*_, (N_*_ − N_G_)/N_*_	the variants of normalized values to characterize the breaking point along the axis of the number of stress cycles;
σ_R_/σ_*_, (σ_*_ − σ_R_)/σ_*_	the variants of normalized values to characterize the breaking point along the stress axis;
*k*	the correlation coefficient;
σ_x_, σ_y_	the standard deviations corresponding to random variables X and Y;
*Cov*(*X*,*Y*)	the covariance, or the average of the products of deviations for each pair of data points;
µ*_x_* and µ*_y_*	the sample means of the compared data sets;
n	the sample size;
K	the reliability of the coefficient of approximation (coefficient of determination);
*I*(*X*,*Y*)	the cross-entropy value;
*N_xy_*	the total number of cells in the merged space;
*N_x_*	the number of cell projections onto space *X*;
*N_y_*	the characteristic spread of all data along the *Y* axis;
recalculated fatigue parameters:	
σ_re_	recalculated strength;
*N*_re_	recalculated fatigue life;
tgα*_W_*_re_	recalculated inclination angle.
